# Modern Anchorage Systems in Orthodontics

**DOI:** 10.7759/cureus.31476

**Published:** 2022-11-14

**Authors:** Sakshi S Umalkar, Vikrant V Jadhav, Priyanka Paul, Amit Reche

**Affiliations:** 1 Public Health Dentistry, Sharad Pawar Dental College and Hospital, Datta Meghe Institute of Medical Sciences, Wardha, IND; 2 Orthodontics and Dentofacial Orthopaedics, Sharad Pawar Dental College and Hospital, Datta Meghe Institute of Medical Sciences, Wardha, IND

**Keywords:** orthodontics, inter-maxillary force, temporary anchorage devices(tads), mini-implants, micro-implants, anchorage

## Abstract

Anchorage has been a vital topic since the origin of orthodontics. In the orthodontic process, gentle, constant pressure is applied to the teeth that need to be moved against the other teeth, which serve as the anchoring unit. The anchoring teeth must be completely stable. The introduction of temporary anchorage devices to the orthodontic field has made it possible to overcome conventional anchorage and its limitations. Mini implants have widened the horizon of the orthodontic field. Skeletal anchorage has, to a large degree, replaced conventional anchorage in a situation where anchorage is considered either critical, insufficient, or likely to result in undesirable side effects such as vertical displacements generated by intermaxillary force systems. Over the last few years, anchorage control with mini-implants has acquired plenty of significance in the clinical management of orthodontic patients. The mode of anchorage facilitated by these implant systems has a unique characteristic owing to their temporary use, which results in a transient, albeit absolute anchorage. The foregoing properties, together with the recently achieved simple application of these screws, have increased their popularity, establishing them as a necessary treatment option in complex cases that would have otherwise been impossible to treat. This comprehensive review aims to present and discuss the historical view, clinical uses, benefits, and drawbacks of the mini-screw implants used to obtain a temporary anchorage for orthodontic applications. Topics to be discussed include classification, types and properties, types of screw, head, and thread, their clinical applications, sites, and placement method selection.

## Introduction and background

For the correction of different types of malocclusions, the secured anchorage is the primary requirement. For the past few decades, anchorage requirements were provided by intraoral (teeth) as well as intramaxillary appliances [1]. However, these treatment modalities might not be able to achieve adequate anchorage control. To highlight these limitations, temporary anchorage devices were introduced in the orthodontic field. A temporary anchorage device (TAD), as defined by Cope JB et al. [2], is “a device that is temporarily ﬁxed to the bone to enhance orthodontic anchorage either by supporting the teeth of the reactive unit or by obviating the need for the reactive unit altogether and which is subsequently removed after use” [2]. Bone-based anchor units are made up of small pins and tiny plates that are also referred to as "transient anchorage units" or temporary anchorage devices (TADs). Presently, TADs are considered to be the commonest method for treating anchorage in orthodontics with negligibly invasive techniques and cost-effective benefits [3]. Cope JB et al. realized that a review of published studies in the orthodontic literature has captured the potential of overcoming anchorage drawbacks through the use of momentary anchorage devices [3]. TADs can be located via transosteal, subperiosteal, or endosteal [3].

## Review

Historic outlook

According to historical documents, implants were originally framed up around 600 AD. A portion of the jaw was used as an implant to duplicate three lower incisors in Mayan population stated by Sana S, Manjunath G [4]. In 1809, Maggiolo [5] documented the development and implantation of gold roots to stabilize the roots. Maggiolo surgically implanted a gold prosthetic tube into a fresh extraction site. This site healed, and a veneer was placed. Nevertheless, there was severe infection of the gum tissue as a result of the technique, reviewed by Abraham CM [5] in his article on the brief viewpoint on dental implants, their surfaces, and their treatment plan. In 1891, Hartman [6], a dentist, advocated the use of metal pins to secure dentures to the jaw. In a 1909 invention titled "Configuration for Manufactured Teeth," Greenfield EJ [7] anticipated a retainer-based denture that would be placed in a hole, as in the alveolar bone. Greenfield EJ again used a "24-gauge iridium-platinum sphere bonded with 24-karat golden metal as a synthetic base in 1913, and it fit the round opening created for one in the patient's mouth. Alvin and Moses Strock [5], two siblings, worked with orthopedic pin fittings constructed of Vitallium in the 1930s (chromium-cobalt alloy). They thoroughly studied how doctors efficiently inserted prosthetics in the hip bone and then placed them in both humans and animals to replace specific teeth. The Vitallium screw offered anchoring and strength for the misplaced dental prosthesis. These siblings were recognized for their efforts in identifying a nontoxic material for use in mammalian teeth. The Strock brothers have also been regarded to be the first to successfully install the very first endosteal (in the bone) implant. Alvin Strock not just experimented with metallic implants, but he also pioneered the importance of antibacterial drugs for the cure of periodontitis, such as Vincent’s angina. P.B. Adams [5] copyrighted a cylinder-shaped Osseo integrated device with an implant attached to a mucosal band and a therapeutic lid inside and outside it in 1938. Gains forth and Higley [6] investigated in 1945 the possibility of anchoring orthodontics to the underlying skeletal part by inserting Vitallium pins into a canine phalanx to distalize a maxillary canine. Around 1998, Costa and co-workers [8] worked on 2 mm titanium mini screws for dental anchoring. The screws were individually driven within the tissue with a torque wrench without generating a flap and were loaded immediately. During the commercial experiment, two of the 16 mini screws used failed to hold and got misplaced before the completion of therapy.

**Figure 1:**
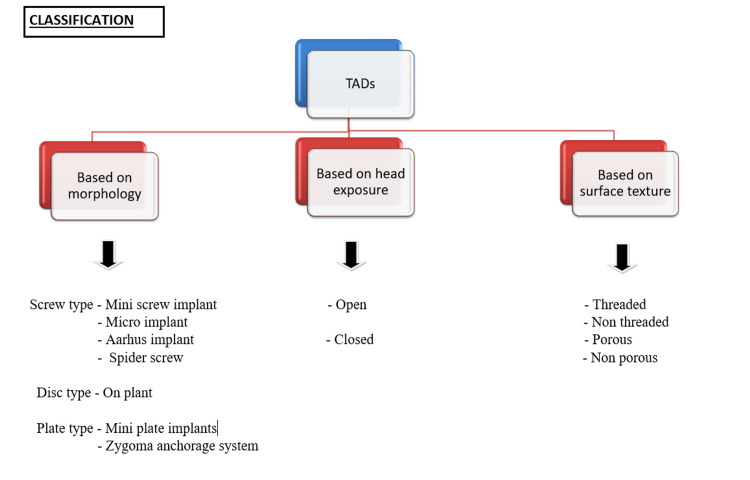
Classification of temporary anchorage device Author Credits: Sakshi Umalkar Source [9]

**Figure 2:**
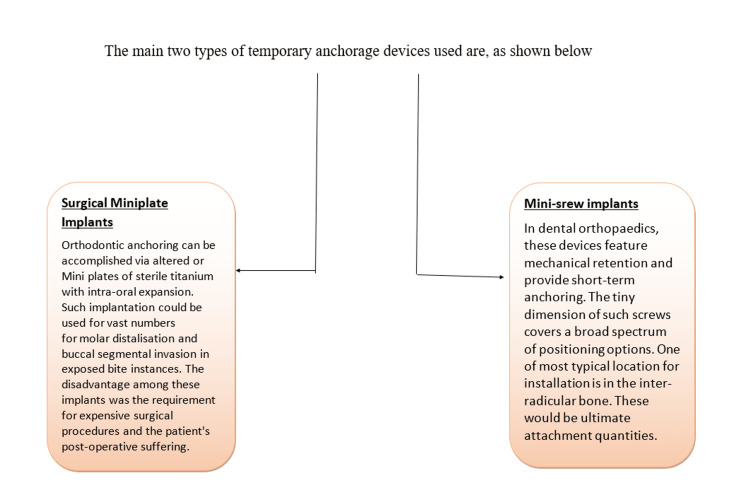
Subdivision of temporary anchorage device Author credits: Sakshi Umalkar Source [10,3]

Figures 1, 2 show the classification of temporary anchorage devices according to different types.

Indications

Definitive indicators for anchorage systems are unclear. The majority of the available publications were documented cases in which novel techniques were presented as options for other fixation systems, such as in extraction situations where implantation was used instead of headgear [10,11].

Some of the indications are: I) Provision of anchorage, which includes en-mass retraction [12] and canine retraction. II) Bimaxillary protrusion [13] includes molar distalization in class 1 molar correction, intrusion of anterior teeth [14], intrusion of posterior teeth, and unilateral intrusion to correct occlusal cant. III) Skeletal orthopedic correction of class 2 and class 3 malocclusions [15]. IV) Other miscellaneous indications include providing attachment for artificial teeth in hypodontia cases and to provide intermaxillary fixation (IMF) during orthognathic surgeries [11].

Provisional anchors also have been placed in advanced dental treatments, as reviewed by Sofie Hoste et al. [16], aiming both the objective of bringing an overview of the fundamental and regional risk factors during TAD implementation and the demands for placing it, as well as to highlight the orthodontic evidence of different TADs units.

Risk factors for placement of TADs

Smoking

Patients who smoke have higher failure rates along with greater loss of marginal bone around titanium implants. Poor wound healing is seen in heavy smokers who take more than 10 cigarettes per day [17]. If periodontal biofilm cannot be controlled, some writers recommend quitting smoking at a minimum of one week before and after dental implant surgery. Because orthodontists have frequent interaction with teens, one author believes dental orthopedic surgeons can play an essential role in deterring children from smoking [18,19].

Age

Insertion of TADs in the median region of the palate should be delayed until adulthood or at least until the mid-palatal suture has calcified. King and co-workers reported that in adolescents, the bone in the paramedian regions of the palate was between 2.1 mm and 7.5 mm thick. They recommended that the thickness of the palatal bone should be determined with a cone beam computed tomography before placing an implant or mini screw. In conclusion, temporary anchorage device should be placed carefully in the mid-palatal area, as there may be insufficient bone to sustain the device [20].

Medications 

Some medications that may have an impact on wound repair or gingival condition should be considered prior to implanting TADs in patient populations. Bisphosphonates, immune modulators, epileptic drugs, anti-aggregation drugs, and blood thinners are illustrations of pharmaceuticals that may cause a TAD to fail [21].

Gingivitis

Periodontitis patients should also have their periodontal status addressed prior to orthodontic therapy and TAD insertion. Because oral inflammatory response is one of the major causes of TAD failure, it is critical to encourage TAD patients to practise good dental hygiene. A single tufted brush for cleaning the TAD is a helpful tool [22,23].

Bone Quality

Osseointegration does not occur in TADs. The maximal load for a non-osseointegrated implant is directly proportional to the surface area of an implant that interacts directly with the surrounding bone. In these circumstances, primary stability is determined by the quality or density of a bone. A TAD obtains its primary stability from mechanical retention and can support immediate orthodontic loads. Thick dense, cortical bone offers better mechanical locking for the implant as compared to thin, cancellous bone. Patients with higher mandibular plane angles have significantly thinner buccal cortical bone. The mandibular plane angle may influence the thickness of cortical bone and, hence, the stability of TADs. Hence primary stability of a mechanically retained device depends mainly on the geometric design of implants, bone quality, and its insertion technique [24].

Radiation Therapy

Hyperbaric oxygen treatment should be taken into consideration to improve wound healing in TAD sites in patients receiving radiation therapy. Only the vascular components of the healing tissue can benefit from this treatment. Following the end of radiation, the cellular components renew naturally. Radiation exposure treatment causes early and advanced tissue changes, as well as substantial effects on bone interstitial cells. Furthermore, there has been a reduction in the bone-to-implant interface, cartilage destruction, fibrous formation, and peripheral arterial diseases (osteoradionecrosis). The ultimate consequence is frequently hypocellular, hypovascular, and hypoxic tissue that is resistant to physical or medical shocks [25].

Orthodontic Considerations of TADs

Retraction of Anterior Teeth

Retraction is the most specific technique used while achieving space closure in orthodontics. Shirck et al., in a survey conducted in 2011, found the most frequent use for TADs is anterior retraction in cases in which bicuspids have been extracted or the occasional case with generalized spacing where anchorage is considered. Retraction of the anterior teeth with TADs can be performed in two general ways. In the first, called indirect anchorage, the traditional teeth comprising the anchorage or reactive unit are tied to the TAD, i.e., the unit to be moved is not attached directly to the TAD. With this approach, traditional orthodontic biomechanics may be utilized without anchorage loss. The second approach is called direct anchorage. In this case, the active unit is attached to the TAD and bypasses anchorage to the other teeth. When using this method, clinicians must exercise great caution with regard to biomechanical principles [26].

The strategy used in retraction mechanics must be based on a thorough diagnosis and treatment plan in accordance with the unique requirements of every individual. The two most popular mechanics for anterior retraction are two-step retraction and en-masse retraction. In two-step retraction, retraction of canine teeth is done, followed by retraction of all four incisors, and en-masse retraction involves retraction of all six teeth [27].

As reviewed by Al-Sibaie S et al. [28], using a simple randomization technique, 56 patients (35 males and 21 females) were randomized and divided into two groups to evaluate treatment outcomes between the sliding en-masse retraction of upper anterior teeth supported by mini-implants and the two-step sliding retraction technique employing conventional anchorage devices. Evaluation of anchorage loss using trans-palatal arch (TPAs) with an en-masse retraction of the upper anterior sixth teeth has been found with a minimum amount of anchorage loss, so a decision was made in this study to avoid en-masse retraction of anterior sixth teeth and employ a two-step sliding mechanism hoping to reduce anchorage needs and obtain a molar movement similar to what would be expected in the mini-implants group. But to ease the mechanics and shorten the period of treatment, en-masse retraction using mini-implants is considered a better alternative to the conventional two-step retraction with TPA anchorage and may be a better choice when planning an extraction-based treatment for patients with moderate to severe upper dentoalveolar protrusion.

In a study made by Ghaith MF Al-Imam et al. [29] to assess the effectiveness of the flapless piezocision procedure in accelerating the retraction of upper incisors, the piezocision procedure was found to be effective in accelerating the retraction of four upper incisors, reducing the retraction time, preserving anchorage and enhancing root torque control during retraction.

Park YC et al. [30] delineated a segmental approach employing a palatal appliance connected to two mid-palatal mini implants. He splinted six anterior teeth palatally. The teeth were pulled backward 6.1 mm via an extension arm connected to the palatal appliance with an elastomeric chain. This approach might have reduced the time; however, proved the technique sensitive. The C-implant, given by Chung [31], a kind of mini-implant, has been claimed to achieve its stability by osseointegration and mechanical retention in a 14.5-year-old girl with severe bidento-alveolar protrusion. C implant or micro-implant was placed in the alveolar bone in all four quadrants to provide anchorage for en-masse retraction without even the help of banded or bonded molars. A successful retraction was also achieved.

Protraction of Posterior Teeth

In this clinical scenario, posterior teeth are protracted anteriorly to avoid a need to place an implant and a lifetime of maintenance for a young patient. One of the promising uses of TADs for protraction occurs when a primary second molar is lost, and there is no second bicuspid to replace it. Whether or not teeth are missing in the opposing quadrant will dictate the eventual molar relationship. In the example shown, both the maxillary second bicuspid and mandibular second bicuspid are missing, and a TAD was used for direct anchorage to protract the mandibular first and second molars [32].

*The* *Intrusion of Molar or Posterior Arch Intrusion*

In conjunction with the prosthodontic replacement of teeth, it is often necessary to intrude hyper-erupted unopposed teeth in an opposing arch. Often, teeth can be restored to an appropriate occlusal plane without a reduction in crown height or endodontic therapy before placing a bridge or implant in the opposing arch. It is also a useful procedure in correcting occlusal cants, as well as the intrusion of posterior teeth for open-bite correction [32].

Distalization of Molars

There are several ways to utilize TADs for molar distalization for a Class II dental correction. Currently popular is the use of a palatal TAD(s) attached to a transpalatal arch that is bonded to the second or first bicuspids. The palatal approach has become more popular due to the excellent bone stock found in the para-sagittal area in the bicuspid region. Class II correction appliances, as well as fixed appliances, are compatible with TAD anchorage as well [32].

Gelgör et al. [33] delineate higher molar distalization in 25 adolescent subjects with skeletal category I and dental class II malocclusions. In every patient, he used mid-palatal screws (IMF Stryker, Leibinger Germany) connected to the distalizing appliance, which consisted of a trans-palatal arch soldered to tooth bands. After a brief healing period, Nickel-Titanium coils were fitted between the bicuspids and then in the posterior teeth. They later reported that upper incisors were proclined, the primary molars developed a light distal rotation, and the upper premolars turned mesially, probably because of the pliability of the trans-palatal arch (TPA). Similarly, Park HS et al. [34] investigated the distal movement of posterior teeth in 13 patients. He placed eleven micro-screws within the jaw and four micro-screws in the maxilla. He reported a hit rate of 90%. 

Anterior Intrusion for Deep Bite Correction

These devices are very useful (using either a direct anchorage or an indirect anchorage) for the intrusion of anterior teeth for correction of a deep overbite. This is particularly helpful in patients with excessive gingival display and maxillary incisor display with the lips in repose. Typically, TADs can be used with direct anchorage to the maxillary incisor segment, or an indirect anchorage can be utilized when an intrusion auxiliary arch is utilized for incisor intrusion [32].

Additional Uses

One of the most promising uses of TADs is with expansion appliance anchorage in patients who were once thought to be past the age at which the palate can be expanded. Sutural separation has been documented in these patients at a more advanced age than was once thought possible - again expanding the boundaries of traditional orthodontics [32]. Other uses for TADs include uprighting molars, appliance anchorage, eruption of impacted teeth, assisting in tooth movement to shift maxillary and mandibular midlines, and as attachments for elastics in condylar fractures in young patients (especially those in whom all permanent dentition has erupted), essentially replacing arch bars and their accompanying undesirable sequellae. 

**Figure 3:**
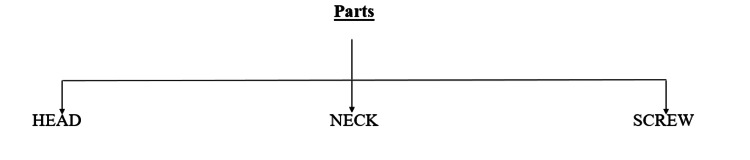
Parts of mini screw implants or (TADs) Author Credits: Sakshi Umalkar

Parts of temporary anchorage devices are the head, neck, and screw thread. Figure 3 shows various parts of TADs.

Head

**Figure 4:**
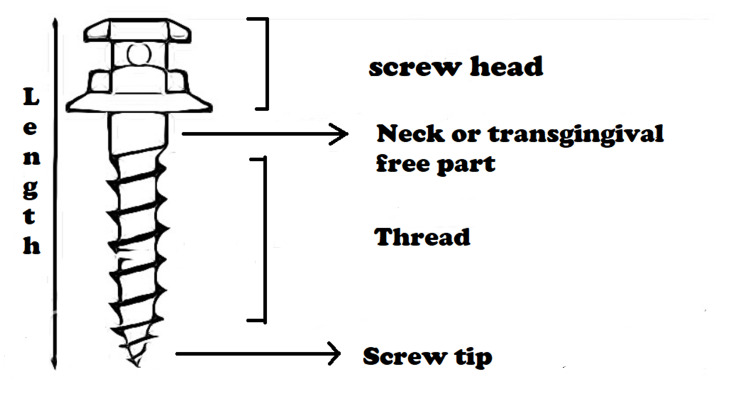
Labeled diagram of parts of mini-screw implant Author Credits: Sakshi Umalkar

It is the orally accessible part of the mini-screw implant that holds the springs and rubber bands in place. It has a special spot that is designed in such particular ways that engage the mini-screw driver for implant placement. For acquiring varieties of anchorage and avoiding soft tissue irritation, different types of heads are available. Button-like design with a sphere or double sphere-like shape or a hexagonal shape is found to be the most commonly used design. For direct anchoring, a hole of 0.8 mm diameter in the head or neck of the screw is typically preferred. Moreover, a loop and collared neck design are also available, which can be used in both types of anchorages. This part is specific to specific manufacturers. The screw head also comes with a hole or collar to give different attachments [35-37]. Figure 4 illustrates the labeled diagram showing parts of mini-screw implants. 

Neck

The neck of the screw, also known as the transmucosal portion, passes through the mucosal part and secures the screw to the head. Variable neck lengths are provided in a way that suits different mucosal thicknesses. To prevent plaque or debris accumulation over the neck surface, it must be smooth and well-polished. Most of the failures of implants at this point are due to its crucial junction with mucosa, as various inflammation issues start from this part of the implant [37].

Screw/Thread

This part provides retention by being embedded in cortical or medullary bone. The stresses created throughout the insertion and also the quantity of insertion torsion needed are determined by the depth and angle of the cutting edge [37]. The thread of the screw around the shank or main body of the TAD has a cutting edge that facilitates insertion. The number of stresses and torque required for the insertion of TAD is determined by none other than the cutting edge and the angle. Thread design may be conical as in mini-screws or parallel tapering at its end point only as in implants in orthodontics. The length of TAD is defined by its thread's body length, ranging from 5 and 12 mm in length according to anatomic requirements. The whole mini-screw length can be judged by the length of the whole parts of the implant, i.e., head, neck, and screw. Screw pitch is a term used for the distance between two threads. Threads positioned wide apart have a high pitch, while threads positioned nearer have a low pitch. A screw having a higher pitch than normal is inserted quickly and fast [37].

Sites for placement

When absolute anchoring of the implant is necessary, mini-screws are employed instead of standard appliances such as lingual arches. The most prevalent locations for mini-screw anchorage system (MAS) insertion in the upper jaw or maxilla are the incisive fossa, canine fossa, infra-zygomatic ridge, pre-maxillary region, or mid-palatal region. The most typical locations for mini-screw installation in the lower jaw or mandible are symphysis, canine fossa, anterior external oblique ridge, retro-molar area, or sub-maxillary fossa. TADs can be sited in extra alveolar bone, but this will cause force on the center of resistance of the tooth. Such implants, however, will get entrenched in the moveable alveolar mucosa, which can be prevented by utilizing trans-mucosal implants. The buccal or lingual inter-radicular gaps between the second bicuspids and molars in both arches, and buccal spaces between the upper anterior in both arches, are considered as most beneficial areas in our experience [37]. With the help of studies, it has been shown that TADs in the maxilla will be placed safely in the apical portion and the tuberosity region but then were avoided due to reduced bone thickness in this region [38].

Implant standards

Implant Material 

The metal should be non-destructive and biodegradable, with exceptional physical and mechanical properties, including tolerance to tension, deformation, and rusting. The material which is often used can be of three varieties; the first is bio resistant, which contains materials like stainless steel and chrome-cobalt alloy. The second one is bio-inert includes materials like titanium and carbon. And the third one is biologically active materials, including hydroxyapatite and oxidized aluminum with ceramics. Due to its properties (without allergic or immunological reaction and tumor growth), titanium is considered an excellent material and is often used [39].

Implant Dimensions

Implant accessories should accomplish primary stability and face up to mechanical loads. The whole bone-implant interface determines the most load. The simplest dental medicine anchorage device has not, however been recognized. Completely different sizes of implants are available to extend anchorage, from "small implants" (6 mm in length, 0.6 mm of radius) to "traditional implants" used in dentistry (6-15 mm length, 1.5-2.5 mm radius) [39].

Implant Form

The amount of bone-to-implant contact area available for stress transmission and early stability is determined by its form and shape. The design should reduce the surgical burden while providing adequate primary stability. In investigations, the degree of surface roughness has been associated with the degree of osseointegration [39].

Techniques for placement of TADs

Mini-implants can be of two types: self-tapping and self-drilling. In self-tapping screws, the mini-screw is inserted into the bone tunnel made by drilling, causing it to hit when the implant is inserted. This approach is used while employing mini screws with tiny diameters. In self-drilling screws, the mini screw is pushed into the bone without drilling. If we need to use mini screws with a larger diameter (1.5mm), we can use this method. Physicians have recently used 3D-cone beam computed tomography and stereolithographic methods to design surgical guides [39]. 

*For self-tapping screws*, pre-drilling is performed with any low amount of local/topical anesthetic agent (ideally by an oral surgeon). Soft tissue is removed upon entering the safe position using a soft tissue punch, and a dig has been formed with a pilot rotatory tool at about a speed of 1000 RPM or less. The pilot hole should be no more than 2 to 3mm deep and 0.3mm narrower than the screw in diameter. The implant is then assembled using a suitable screwdriver [37,40]. Self-drilling screws feature precisely shaped tips and sharp flutes that allow them to be driven into bone without pre-drilling, lowering the risk of damage to the root of the tooth, tooth germs and nerves, as well as the necrosis of the thermal bone and the fracture of the drill bit. However, if the thickness of the corticulated bony surface is above 2mm, a pilot hole is needed to be drilled, which in turn can cause the bending of the tip of the screw [37,41]. 

Table 1 differentiates between self-drilling and self-tapping screws.

**Table 1:** Difference between self-drilling and self-tapping screws Author Credits: Sakshi Umalkar Source [37]

Self-Drilling Screw	Self-Tapping Screw
Requires cutting tip	No need of cutting tip
Pilot hole is not necessary	Pilot hole is being drilled as same length as of implant
May cause loss of tactile sensitivity	No evidence of loss of tactile sensitivity
There may be bone compression, patient discomfort and root resorption after placement	Can be placed without any difficulty and minimal tissue damage after pilot hole is being placed

Benefits and Drawbacks of TADs in Orthodontics

Table 2 shows the benefits and drawbacks of TADs. 

**Table 2:** Benefits and drawbacks of TADs Author Credits: Sakshi Umalkar Source [42]

Benefits	Drawbacks
Makes the sufferer feel better.	The necessity for invasive surgery, irritation of mucosal membranes, and injury to roots or neurovascular bundles.
Minimal emphasis on patient consent.	Not ideal in the mixed dentition stage.
Versatile placement, i.e., buccal or palatal or maxillary or mandibular.	It is expensive, and there can be screw fracture mobility after removal.
The main advantage of these implants is that they allow many teeth to be moved without losing attachment.	It is technique-sensitive and possible chances of infection are present.
They can be used in regions where natural anchoring or traditional orthodontic equipment are impractical, such as edentulous gaps in the alveolus of either arch, the palate, the zygomatic process, the posterolateral region, and the suspensory ligament.	Various implantation sites are having varying anatomical characteristics.

Complications

Complications while insertion may include trauma to a periodontal ligament or root tooth due to a change of angle of insertion. There could be damage to the nerve or slippage of mini screw, fracture, nasal and sinus perforation also. Soft tissue complications include aphthous irritations, additional soft tissue growth on the head of the mini screw along with auxiliaries, soft tissue inflammation, and infection within the implants. Two issues that might arise after removal include mini-screw fracture and incomplete osseointegration [43]. Other potential complications are the breakage of the screw within the bone and inflammation around the site. 

## Conclusions

Over the past few decades, conventional anchorage, which is considered either critical or insufficient, has been replaced by skeletal anchorage with minimal invasiveness and desirable properties. These skeletal fixtures would make the outcome more predictable and satisfying for orthodontists and patients. The orthodontist can use the temporary anchorage device (TAD) to help with a variety of issues that arise when tooth displacement occurs. Despite drawbacks such as root damage, implant infections, and failures in implants, TADs have a significant role in orthodontics due to the benefits of simpler placement and withdrawal, instantaneous placement, and appropriate anchorage. A detailed understanding of the elements that influence micro implant success can aid in obtaining targeted treatment outcomes with little patient chair-side time.
